# Examination of Triacylglycerol Biosynthetic Pathways via *De Novo* Transcriptomic and Proteomic Analyses in an Unsequenced Microalga

**DOI:** 10.1371/journal.pone.0025851

**Published:** 2011-10-17

**Authors:** Michael T. Guarnieri, Ambarish Nag, Sharon L. Smolinski, Al Darzins, Michael Seibert, Philip T. Pienkos

**Affiliations:** 1 National Bioenergy Center, National Renewable Energy Laboratory, Golden, Colorado, United States of America; 2 Computational Science Center, National Renewable Energy Laboratory, Golden, Colorado, United States of America; 3 Biosciences Center, National Renewable Energy Laboratory, Golden, Colorado, United States of America; 4 Energy Sciences Directorate, National Renewable Energy Laboratory, Golden, Colorado, United States of America; Rutgers University, United States of America

## Abstract

Biofuels derived from algal lipids represent an opportunity to dramatically impact the global energy demand for transportation fuels. Systems biology analyses of oleaginous algae could greatly accelerate the commercialization of algal-derived biofuels by elucidating the key components involved in lipid productivity and leading to the initiation of hypothesis-driven strain-improvement strategies. However, higher-level systems biology analyses, such as transcriptomics and proteomics, are highly dependent upon available genomic sequence data, and the lack of these data has hindered the pursuit of such analyses for many oleaginous microalgae. In order to examine the triacylglycerol biosynthetic pathway in the unsequenced oleaginous microalga, *Chlorella vulgaris*, we have established a strategy with which to bypass the necessity for genomic sequence information by using the transcriptome as a guide. Our results indicate an upregulation of both fatty acid and triacylglycerol biosynthetic machinery under oil-accumulating conditions, and demonstrate the utility of a *de novo* assembled transcriptome as a search model for proteomic analysis of an unsequenced microalga.

## Introduction

Oleaginous microalgae produce substantial amounts of neutral lipids, primarily comprised of triacylglycerides (TAGs) of favorable fatty acid chain length, making them an ideal feedstock for conversion to biodiesel or renewable diesel and jet fuel [1], [2], [3]. Many of these species can also grow rapidly under a large range of environmental conditions, such as varied light intensity, temperature, and nutrient availability [1], [2]. Microalgae are also capable of growth on non-arable land using a variety of water sources, including fresh, brackish, saline, and waste water [1], [4], [5]. At current production levels, oleaginous microalgae also have the potential to produce 1–2 orders of magnitude more oil per acre than soybeans, the most common US oilseed crop (∼50 gal acre^−1^ y^−1^) [6]. The Department of Energy funded an almost 20-year effort, known as the Aquatic Species Program (ASP), aimed at developing algal biofuels. The ASP was terminated, however, in 1996 because the projected cost of algal biofuels could not compete at that time with the low price of petroleum. Now, after more than a decade, microalgae have again risen to international prominence for their potential to contribute to the global liquid fuel demand. However, despite the great promise of algae-based fuels, our understanding of algal lipid metabolism, particularly the regulation of biosynthetic pathways of fatty acids and TAGs, as well as the metabolism affecting carbon partitioning, is still largely inadequate. Furthermore, the lack of available genome sequence information limits the development of basic biological understanding required for strain improvement of unsequenced microalgae.

Rapidly developing post-genomic, systems biology approaches such as transcriptomics, proteomics, and metabolomics have become essential for understanding how microorganisms respond and adapt to changes in their physical environment. The application of such high-throughput approaches could greatly accelerate the commercialization of algal-derived biofuels by providing the framework for hypothesis-based strain improvement programs, built on an improved fundamental understanding of the specific pathways and regulation of networks involved in algal oil production. However, a systems biology approach to investigating oleaginous microalgal metabolism remains relatively unexplored especially in unsequenced organisms. To date, the nuclear genomes of only about ten eukaryotic microalgae have been sequenced (http://genome.jgi-psf.org), and though a few of these strains may have some properties of interest for algal biofuels, most, if not all of them, were chosen to be sequenced for reasons other than biofuel production.

“Higher level -omic” analyses – transcriptomics, proteomics, and metabolomics – of microalgae have largely focused upon those species with sequenced genomes (for an extensive review, please refer to Jamers, 2009). For example, the transcriptome of the model green alga, *Chlamydomonas reinhardtii*, was recently characterized under nutrient-replete, anaerobic H_2_-producing, and sulfur-depleted growth conditions [7], [8], [9]. Benning and coworkers have also extensively examined the nitrogen-deprivation stress response and its effects upon lipid accumulation in *C. reinhardtii*, through comparative transcriptomics and lipid droplet proteomics [10], [11]. However, the best cellular lipid accumulation noted for this organism is only ∼20% in wild type cultivars, though ∼40% total lipids have been reported for starchless mutants [12], [13]. Many unsequenced wild-type strains have been reported to make more than 50% total lipids under these same growth conditions [1].

It is likely that the dearth of microalgal genomics data has dissuaded biologists from pursuing higher-level systems biology analyses of unsequenced yet potential commercially relevant oleaginous microalgae. Transcriptomic analyses focused upon elucidating specific metabolic pathways have, up until recently, employed microarrays generated from cDNA libraries, or sandwich hybridization assays, as opposed to utilization of the high-throughput, massively parallel, cDNA-sequencing techniques currently available for global transcriptome analysis [14], [15], [16]. On the other hand, Rismani-Yazdi et al. [17] just reported 454 pyrosequencing to carry out the first *de novo* transcriptomic sequencing and annotation of the unsequenced microalgae *Dunaliella tertiolecta*. This work marks a meaningful advance in the pursuit of higher-level systems biology approaches with unsequenced, oleaginous microalgae, and demonstrates the capability of using transcriptomic data to identify pathways and targets of interest for metabolic engineering and functional genomic analyses in non-model microalgae.

Despite its power, transcriptomic analysis does not adequately define the control points for metabolic regulation. This is especially true in algae where post-transcriptional regulation is not well understood. For example, translational regulation of chloroplast gene expression occurs in a number of microalgae and higher plants [18], [19], [20], [21], [22], [23]. Thus, a more complete systems biology analysis is required to provide useful hypotheses for strain improvement strategies. Proteomics can bring us closer to this goal, but the availability of proteome data from unsequenced microalgae is also sparse. Such proteomic analyses have typically targeted specific cellular components (sub-proteomes such as chloroplasts) and yielded relatively low orthologous identification rates [16], [24], [25]. For example, Wang et al. [25] employed a cross-species protein identification strategy in order to examine the proteome of the *Haematococcus pluvialis* cell wall. For the identification of proteins with low sequence identity, a conserved motif and domain strategy was also implemented. They observed that approximately one half of the proteins examined failed to be recognized in protein databases. This was attributed to amino acid substitutions and/or post-translational modifications, both of which dramatically reduce the probability of cross-species proteomic identifications. Such analyses underscore the need for increased sequence information on diverse microalgae. As we present in this communication, utilization of a *de novo* sequenced transcriptome can provide the necessary sequence information to pursue such proteomic analyses.

After considering the fact that of the over 40,000 species of microalgae identified to date, fewer than a dozen microalgal genome sequences are available, it is not surprising that elucidation of the key pathways and networks regulating lipid accumulation remains limited [1]. An integrated systems biology examination of these organisms will be critical in order to understand the unique, strain-specific mechanisms of lipid accumulation, and to develop strategies required to engineer improved strains with enhanced lipid production. Furthermore, genetic engineering of unsequenced strains will require identification of unique promoter and untranslated region (UTR) sequences for targeted overexpression or silencing of target genes [22]. Transcriptomics and proteomics cannot provide complete sequence data for promoters and UTRs, but can identify genes with desirable expression patterns, thereby directing strain-engineering strategies to a small region rather than the entire genome. And so, with the full potential of transcriptomics and proteomics largely dependent upon genome sequence availability, many promising algal strains have been left unexplored.

The oleaginous green alga, *Chlorella vulgaris*, has been extensively studied due to its relatively fast growth rate and its value as both a food supplement and potential biofuel feedstock. Additionally, *C. vulgaris* has also recently been examined in light of the genus' biomedical relevance, demonstrating anti-oxidant and anti-tumorigenic properties, as well as having value in increasing vascular and immune function [26], [27], [28], [29]. *C. vulgaris* also accumulates >50% lipid under nutrient-deplete conditions, with a favorable fatty acid profile for biodiesel production. Finally, many reports have been published describing successful genetic transformation of *Chlorella* cultivars [30], [31]. Taken together, we have concluded that this is an ideal platform to explore algal lipid metabolism and the biosynthetic pathways involved in fatty acid and TAG biosynthesis in oleaginous algae. To date, however, there is no genome sequence available for *C. vulgaris* (although a genome sequence has been published for the presumably related strain, *C. variablis* NC64A [32]), hindering the further development of this organism as a food, fuel and biomedical resource.

We have taken a different approach and set out to demonstrate the utility of bypassing the genome sequencing step by taking advantage of current high-throughput technologies in order to pursue direct, higher-level systems biology analyses. Herein, we have built upon the *de novo* transcriptome sequencing approach, and set out to conduct a comparative global transcriptomic and proteomic study of the microalga, *C. vulgaris* UTEX 395, chosen after screening all ten *C. vulgaris* cultivars in the UTEX Algae Culture Collection for growth rate and lipid accumulation capability, under conditions that induce high oil production. These conditions have been optimized to yield greater than 60% fatty acid accumulation based on dry cell weight when the algae were grown under nutrient-deplete conditions. cDNA from *C. vulgaris* was sequenced using Illumina technology and *de novo* transcriptome assembly was performed using a combination of readily available software and newly generated bioinformatic tools. The proteomic analysis was subsequently undertaken utilizing the assembled *C. vulgaris* transcriptome as a search model. This work marks the first comprehensive proteomic investigation of lipid accumulation in an unsequenced microalga, as well as the first utilization of a *de novo* assembled transcriptome as a search model for proteomic analysis in an unsequenced microalga. Our results indicate that this approach can provide a powerful and effective search model for proteomic analysis. Our efforts demonstrate the feasibility of bypassing the bottleneck of genomic sequencing, opening the door for a comprehensive systems biology examination of other unsequenced oleaginous microalgae.

## Materials and Methods

### Algal strain and culture conditions

*Chlorella vulgaris* strain UTEX 395 was grown in 1L Roux bottles using modified Bold's Basal Media (mBBM) containing: 2.94 mM NaNO_3_, 0.17 mM CaCl_2_, 0.30 mM MgSO_4_, 0.43 mM K_2_HPO_4_, 1.00 mM KH_2_PO_4_, 0.43 mM NaCl, 0.17 mM EDTA, 18 µM FeSO_4_, 0.18 mM H_3_BO_3_, 61 µM ZnSO_4_, 15 µM MnCl_2_, 10 µM MoO_3_, 13 µM CuSO_4_, and 3.3 µM CoNO_3_. Cultures were maintained at 25°C±1°C, with continuous (24 hr) white fluorescent light illumination (200 µE m^−2^ s^−1^). Cultures were supplemented with 2% CO_2_/air and mixed with a magnetic stir bar at 500 rpm. To induce nitrogen deprivation, rapidly growing cells were centrifuged for 5 min at 5,000× *g*, washed once in nitrogen-free mBBM, and resuspended in nitrogen-free mBBM for continued growth. Cell growth was monitored via cell count measurements using a 0.1 mm depth, Hy Lite_hemocytometer (Hausser Scientific). Cultures were inoculated at a cell density of approximately 3.5×10^6^ cells/mL.

### Fatty Acid Methyl Ester (FAME) analysis

Total fatty acid content was determined via transesterification of glycerolipids followed by GC-FID analysis, using a method adapted from Lepage and Roy [33]. Briefly, 50 mL samples of cell culture at 7.85×10^7^ cells/mL (nitrogen replete) and 5.00×10^8^ cells/mL (nitrogen deplete) were harvested via centrifugation for 5 min at 5,000× *g*. Cell pellets were quenched in liquid nitrogen and lyophilized overnight. Approximately 5 mg of dry biomass was suspended in chloroform-methanol (2∶1, v/v), and glycerolipids were transesterified in HCl-methanol (5%, v/v) for 1 h at 85°C in the presence of a tridecanoic acid methyl ester as an internal standard (Sigma Aldrich). Fatty acid methyl esters were extracted in hexane (Sigma Aldrich) at room temperature for 1 hr and analyzed by GC-FID. For all FAME analyses, three replicates were examined.

### Fluorescence Microscopy

Non-polar lipid accumulation was qualitatively examined using epifluorescence microscopy. Cells were harvested from nitrogen replete and nitrogen deplete media, as described above. Cells were treated with 10% DMSO (to increase membrane permeability) and stained for five minutes with 10 µg/ml of the non-polar lipid fluorescent dye, BODIPY 493/503 (Molecular Probes, Invitrogen Corporation). Cells were immobilized on microscope coverslips by mixing with 1% low-melting-temperature agarose (heated to 65°C to solubilize) in a 1∶1 ratio. Images were acquired using a Nikon Eclipse 80i Epifluorescent microscope. Chlorophyll autofluorescence was detected using a 660/50 band-pass optical filter, and BODIPY 493/503 fluorescence was detected using a 525/50 band-pass filter.

### Isolation of mRNA

A 50-mL sample of a nitrogen replete cell culture was harvested via centrifugation for 2 minutes at 3,000 × *g*. Cell pellets were resuspended in 5 mL RNeasy Lysis Buffer (Buffer RLT, Qiagen, RNeasy Plant Mini Kit), frozen in liquid nitrogen, and ground into a fine powder by mortar and pestle on liquid nitrogen. RNA was extracted using a Qiagen RNeasy Plant Mini Kit with glass bead disruption. The lysate was divided among 10 microfuge tubes and eluted into RNase-free ddH_2_0. A Qiagen RNeasy MinElute Cleanup Kit was utilized to concentrate the RNA.

### Isolation of soluble protein fraction

Cells were harvested at cell densities of 7.85×10^7^ cells/mL (nitrogen replete) and 5.00×10^8^ cells/mL (nitrogen deplete) via centrifugation for 2 minutes at 3,000× *g*. Cell pellets were immediately quenched in liquid nitrogen, thawed and solubilized on ice in 2 mL of lysis buffer (50 mM Tris, pH 8.0, 150 mM NaCl, 1 mM DTT, 10% glycerol, supplemented with 1× cOmplete Protease Inhibitor Cocktail solution (Roche Diagnostics Corporation, Indianapolis, IN)). The cells were then sonicated on ice at 4°C, at 90% power setting for 30 seconds×6 cycles, with a one minute cool-down period between sonication cycles using a Braun-Sonic-L ultrasonicator. Lysates were cleared via two cycles of centrifugation at 16,000× *g* at 4°C for 30 minutes, and the supernatants were isolated for use in subsequent proteomic analysis.

### Transcriptome Analysis

Total RNA was first assessed for quality on a Bioanalyzer 2100 using Nano 6000 LabChip (Agilent Inc., Santa Clara, CA). A complementary DNA (cDNA) sequencing library was prepared from the total RNA using a mRNA-seq Sample Preparation Kit (Illumina). Briefly, poly A+ RNA was isolated from 10 µg total RNA using Sera-Mag Magnetic Oligo-dT beads. Purified mRNA was fragmented, annealed to high concentrations of random hexamers, and reverse transcribed. Following second strand cDNA synthesis, end repair, and A-tailing, oligo adapters complementary to sequencing primers were ligated to cDNA fragment ends. Resultant cDNA libraries were size fractionated on an agarose gel by excising 200-bp fragments followed by amplification with 15 cycles of polymerase chain reaction. Clusters were generated from the cDNA sequencing library on the surface of a flowcell in the Cluster Station (Illumina) by bridge amplification. The flowcell was used to perform 56 cycles of sequencing-by-synthesis chemistry in the Genome Analyzer II. The manufacturer's Genome Analysis OLB 1.8 pipeline (Illumina, San Diego, CA, USA) was used to perform image analysis (Firecrest), and base calling (Bustard).

Short nucleotide reads obtained via Illumina sequencing were assembled by the Velvet software [34] to produce error-free, unique contiguous sequences (contigs). The Oases program [35] was then utilized to cluster the contigs in the preliminary Velvet assembly into small groups (loci), and construct transcript isoforms for each of these loci. For the assembly of contigs using Velvet, we chose a *k*-mer length of 25 that maximized the average length of the transcript isoforms that constituted the output from the Oases program. *Chlorophyta* nucleotide sequences were downloaded from the NCBI Gene database and formatted using the makeblastdb program from the standalone BLAST+ program suite in order to obtain a nucleotide database compatible for BLAST analysis. Transcript isoforms were annotated by the local alignment of assembled transcript sequences against this *Chlorophyta* nucleotide database using the standalone NCBI BLAST+ program suite. Nucleotide query sequences of the transcript isoforms were locally aligned against the nucleotide sequences in the database using the nucleotide blast (blastn) program from the standalone BLAST+ program suite, and the results from this nucleotide BLAST+ search enabled the assignment of gene models to these transcripts. The nucleotide blast search was complemented by the local alignment of the six-frame conceptual translation products of the query transcript sequences against a formatted database of *viridiplantae* protein sequences downloaded from the RefSeq protein database using the blastx program. Gene ontology enrichment was performed on the annotated transcriptome and the subset of the transcriptome matching the *C. vulgaris* proteome utilizing the Blast2GO software version 2.4.8 [36].

### Proteome Analysis

Gel-based liquid chromatography-mass spectrometry (GeLC/MS) was employed for comparative shotgun proteomic analysis. 20 µg soluble proteins were separated using one-dimensional SDS-PAGE. Entire lanes were excised from the gel and cut into 40 segments. Gel segments were reduced, alkylated, and tryptically digested robotically, using a ProGest protein digestion station (DigiLab, Inc.) to provide peptide-containing liquid fractions suitable for LC/MS/MS analysis on a Waters NanoAcquity HPLC system interfaced to a ThermoFisher LTQ Orbitrap Velos mass spectrometer. Peptides were loaded on a trapping column and eluted over a 75-µm analytical column at 350 nL/min; both columns were packed with Jupiter Proteo resin (Phenomenex). The mass spectrometer was operated in data-dependent mode, with MS performed in the Orbitrap at 60,000 FWHM resolution and MS/MS performed in the LTQ. The fifteen most abundant ions were selected for MS/MS. For all proteomic analyses, three biological replicates were examined.

In-house Awk and Python scripts were used to convert the annotated transcriptome into a format suitable for input to the proteomic Mascot program [37]. Mascot was used to perform *in silico* six-frame translations of the annotated transcriptome, and the product ion data were searched against the resultant database. Product ion data were also searched against concatenated forward and reverse *Chlorophyta* databases (using all available sequenced microalgae). Databases were appended with commonly observed background proteins (cRAP) to prevent false assignment of peptides derived from those proteins. Mascot DAT output files were parsed into the Scaffold program (Proteome Software) for validation and filtering to assess false discovery rates (FDR), which allowed only statistically significant protein identifications. Scaffold parameters were set to a minimum of 2 peptides per protein with minimum probabilities of 95% at the protein level and 50% (Prophet scores) at the corresponding peptide level in order to ensure <1% FDR. ANOVA statistical analysis and principal component analysis was applied using ArrayTrack [38] in order to identify differential significance between nutrient-replete and depleted samples, as well as between biological replicates. Only those positive protein identifications for which *p*-values less than or equal to 0.05 were obtained were considered statistically significant for the data presented. Data normalization was applied based upon the total number of spectral counts under nitrogen-deplete conditions as described by Zybailov *et al.* [39].

## Results

### Growth and lipid accumulation of C. vulgaris under nitrogen stress

**Figure 1:**
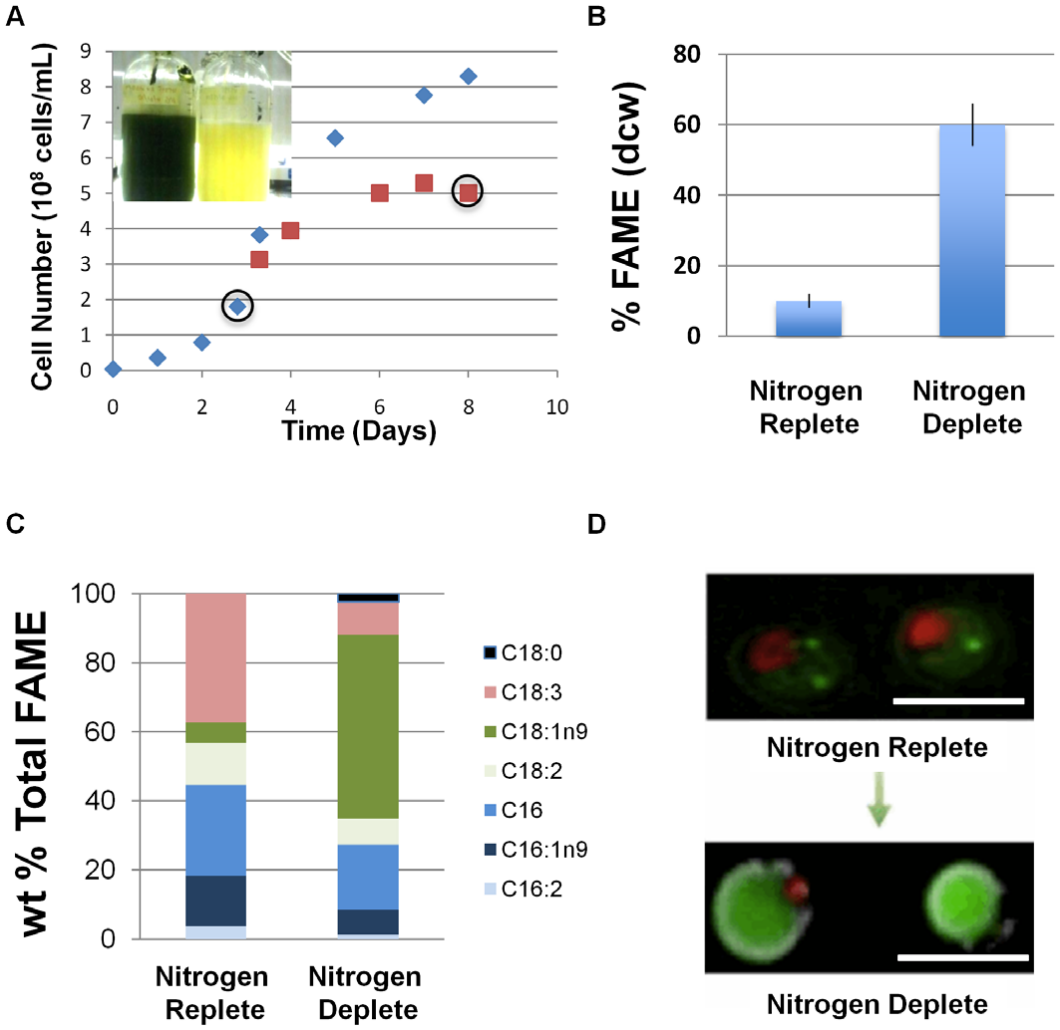
Growth and lipid accumulation properties of *C. vulgaris* under nitrogen limitation. (A) Representative growth curves for *C. vulgaris* cultured photoautotrophically under nitrogen-replete (blue) and nitrogen-deplete (red) conditions. Harvest points utilized for all comparative analyses and images are circled. Inset: cultures grown under nitrogen-replete (green) and nitrogen-deplete (yellow) conditions. (B) Fatty acid methyl ester (FAME) analysis of *C. vulgaris* under nitrogen-replete and nitrogen-deplete conditions. (C) Average fatty acid composition (%, w/w) for *C. vulgaris* under nitrogen-replete and nitrogen-deplete conditions. (D) Epifluorescent microscopy images of BODIPY-stained *C. vulgaris* in nitrogen-replete (top panel) and nitrogen-deplete (bottom panel) medium. Red fluorescence is due to chlorophyll autofluorescence and green fluorescence is due to BODIPY-neutral lipid interaction. Magnification bar (white) equals 5 µm.

*C. vulgaris* UTEX395 was cultured photoautotrophically in small-scale, Roux-bottle photobioreactors under nitrogen-replete and nitrogen-deplete conditions. Cells were harvested from cultures in nitrogen-replete media, followed by buffer exchange to nitrogen-free media, and final harvesting after growth in nitrogen-deplete media in order to obtain mRNA and protein fractions for transcriptomic and proteomic analysis (Figure 1a, circled data points). These harvest points were selected to maximize the differential in lipid accumulation based upon culture sampling throughout the growth curve. Growth under nitrogen-replete conditions led to both faster growth rates as well as higher cell densities compared to nitrogen-depleted cultures, indicative of nitrogen-limitation in the latter. Nitrogen-deplete cultures continued to grow at rates similar to nitrogen-replete cultures for approximately 24 hours before their growth rates declined. It is an interesting observation that cells continue to grow post-nitrogen deprivation. This is likely due to the utilization of internal nitrogen stores, and potentially through the mobilization of nitrogen contained in cell wall chitin (discussed further below; Gerken and Knoshaug, unpublished results). With the decrease in growth rate, the appearance of the culture changed, turning from dark green to yellow (Figure 1a, inset).

We selected harvest points for transcriptomic and proteomic analysis based upon total fatty acid content, as opposed to the expression levels of specific transcripts or proteins, in order to maximize the differential in protein expression specifically with respect to oil accumulation. Lipid accumulation was quantitatively examined via transesterification of glycerolipids into FAME. We chose this method of lipid analysis because in our view, all fatty acids are potential feedstocks for biofuel production and because the conversion of fatty acids and recovery and quantitation of FAMEs provide a much more accurate measure of lipid content than standard gravimetric analyses such as Bligh-Dyer [40]. Under nutrient replete conditions, *C. vulgaris* accumulated 10%±2% fatty acids on a dry cell weight basis (dcw) (Figure 1b). When incubated for ∼5 days in the presence of nitrogen free media, *C. vulgaris* accumulated 60%±6% fatty acids (dcw) (Figure 1b). A major shift in the profile of fatty acids occurs under nitrogen deprivation when compared to nutrient replete conditions. Most notably there is a 9-fold increase in C18:1n9 and a 4-fold decrease of C18:3 (Figure 1c). This result is similar to that observed by Stephenson et al. [41] where *C. vulgaris* cultures grown during nitrogen deprivation accumulated a significant amount of C18:1, while the amount of more highly unsaturated fatty acids (C18:2, C18:3, C16:2) decreased. Conversely, the fatty acid profile observed for *C. vulgaris* under nitrogen deprivation dramatically differs from that observed for *C. reinhardtii* under nitrogen stress where palmitic and linoleic acid (C16:0 and C18:2n9,12), as well as oleic acid (C18:1), significantly increase [42].

We also examined qualitatively the accumulation of neutral lipids under nitrogen replete and nitrogen-deplete conditions by fluorescence microscopy, using the neutral lipid dye BODIPY 493/503 (Figure 1d). Under nutrient replete conditions, representative *C. vulgaris* cells were found to accumulate a few small, discrete green fluorescent lipid droplets. However, under nitrogen deprivation, most *C. vulgaris* cells accumulated large lipid droplets that appear to encompass the bulk of the intercellular space. No notable increase in cell size was observed. This is in contrast to lipid accumulation observed in *C. reinhardtii*, in which there is an increase in the number rather than an increase in size of small lipid droplets [11], [13].

### De novo Transcriptome Assembly and Annotation

**Figure 2:**
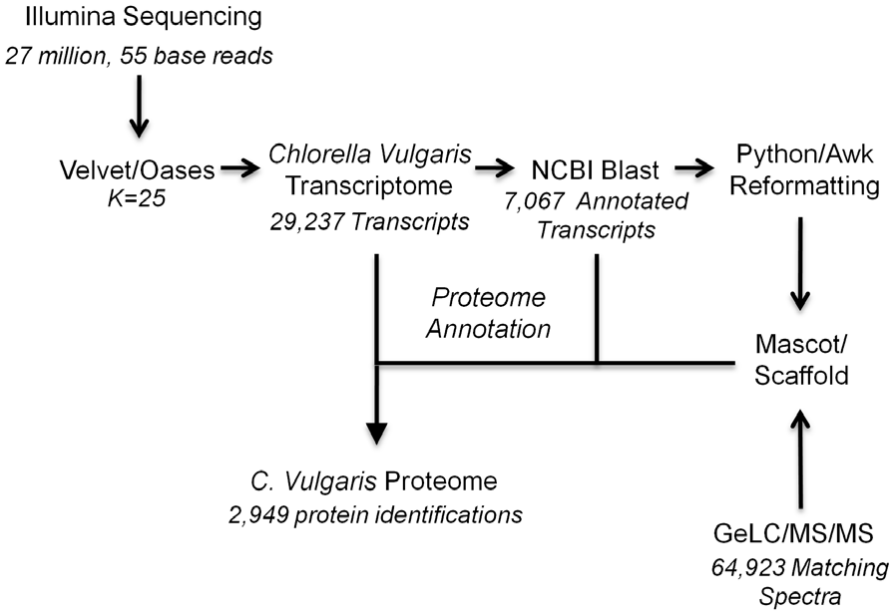
Workflow for *de novo* transcriptome assembly and comparative proteomic analyses.

In order to sequence the *C. vulgaris* transcriptome, a complementary DNA (cDNA) library was prepared from total mRNA, and sequenced-by-synthesis using Illumina technology. Illumina Pipeline analysis produced 27 million, 55-base reads (Figure 2). We were unable to use the *C. variabilis* NC64A and *Coccomyxa* C-169 genome sequences as scaffolds for assembly because the sequence homology was less than 5% for the short reads (http://genome.jgi-psf.org/Coc_C169_1/Coc_C169_1.home.html, data not shown). Therefore, Velvet and Oases program suites were utilized for *de novo* transcriptome assembly. The resultant assembly yielded 29,237 transcripts, with an average transcript length of 970 nt (Figure 2).

Unique transcripts were aligned against all NCBI *Chlorophyta* non-redundant sequences, including whole genome sequences. 7,067 transcripts had hits with significant Expect values (E-values) less than 1e-03, suggesting ∼77% of assembled *C. vulgaris* transcripts are unique amongst available microalgae sequences. As noted by Rismani-Yazdi et al. [17], this identification rate is consistent with previously reported values for *de novo* assembled eukaryotic transcriptomes. Of the corresponding E-values, 315 (4.4%) were scored 0.0, which indicates that the corresponding hits are the best possible BLAST results. The mean E-value was 3.22e-06 and median E-value was 2e-38 (data not shown).

### Isolation and Fractionation of Soluble Proteome

**Figure 3:**
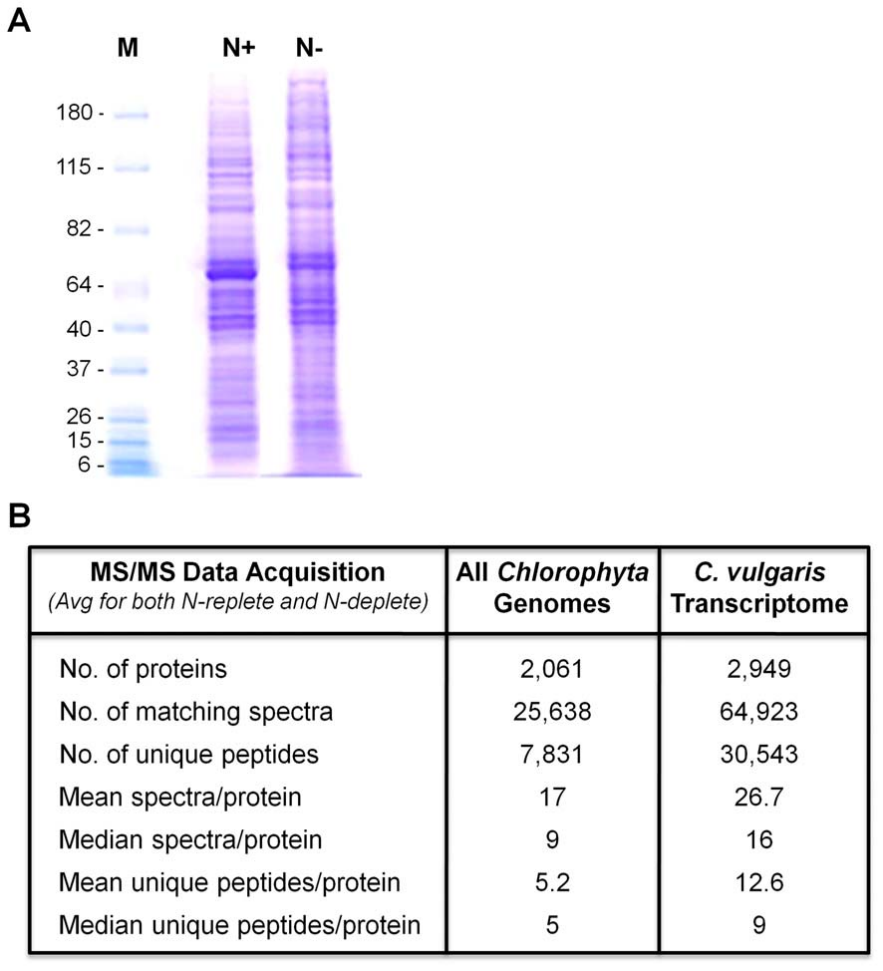
Proteomic Analysis of *C. vulgaris*. (A) One dimensional SDS-PAGE of *C. vulgaris* soluble fraction utilized for comparative proteomic analysis. M, Marker; N+, Nitrogen-replete; N−, Nitrogen-deplete. (B) Mass spectral analysis data for the *C. vulgaris* proteome searched against all available *Chlorophyta* genome databases (left) and the *C. vulgaris* Transcriptome (right).

The cell wall of *C. vulgaris* contains an extensive network of biopolymers, largely composed of chitin and algaenan-like molecules, as well as uronic acid and proteoglycans (for extensive review of *Chlorella* sp. cell wall composition, refer to Takeda, 1991 [43]). The robust nature of this cell wall makes *C. vulgaris* extremely resistant to cell lysis, and in turn, hinders extraction of the soluble protein fraction. Numerous mechanical and chemical cell disruption methods were therefore examined in order to optimize cell lysis and increase sample complexity. Optimal lysis and protein extraction, determined by maximal lysate protein complexity and resolution on SDS-PAGE analysis, was ultimately obtained via multiple cycles of sonication. Increasing the number of sonication cycles improved protein identification rates by more than an order of magnitude (134 proteins from a single cycle, >2,000 proteins from six cycles). No cells remained intact when examined microscopically (data not shown). Beyond six cycles of sonication, protein abundance and complexity did not noticeably improve. Therefore, we utilized six cycles of sonication to maximize lysis efficiency and avoid potential protein degradation caused by excessive sonication treatment. The resulting SDS polyacrylamide gels demonstrate a high sample complexity, with a broad range of highly resolved proteins observed. Differential protein expression patterns between nitrogen-replete and nitrogen-deplete conditions are clearly observed (Figure 3a).

### Proteomic Analysis and Annotation

Proteomic analysis was performed using Gel-based liquid chromatography mass spectrometry (GeLC/MS, Figure 2). In-house Python and Awk scripts were used to annotate the *de novo* assembled *C. vulgaris* transcriptome using nucleotide blast (blastn) results and to properly format the resulting annotated transcriptome for use in Mascot. Each transcript isoform in the assembled transcriptome was annotated using the fasta header of the best blastn hit using Awk and Python codes (Figure 2). Since multiple transcript isoforms corresponding to the same locus or to different loci can have the same top blast hit, multiple transcript isoforms can result in redundant headers, causing errors with the Mascot program. To bypass this problem, multiple occurrences of a given fasta header in the annotated transcriptome file were appended with ascending numbers using a second Python script.

Product ion data was searched against forward and reverse concatenated *Chlorophyta* and six-frame translated *de novo* assembled *C. vulgaris* transcriptome databases using the Mascot search program, using identical search parameters. Searching against *Chlorophyta*, the proteomic analysis identified an average of 1,401 proteins under nitrogen-replete conditions, and 1,347 proteins under nitrogen-deplete conditions, corresponding to 2,061 unique protein identifications between the two conditions (Figure 3b). Searching against the *de novo* assembled *C. vulgaris* transcriptome yielded significantly higher positive identifications. Under nitrogen-replete conditions, an average of 2,312 proteins were identified, and an average of 2,209 were identified under nitrogen-deplete conditions, corresponding to 2,949 unique protein identifications between the two conditions (Figure 3b). Thus, of the 7,067 transcripts identified by blastn search against all *Chlorophyta*, ∼42% were identified in our proteomics analysis. The numbers of matching spectra, unique peptides, mean and median spectra/protein (the average and statistically distributed mid-value of spectral counts, respectively, identifying a given protein), and mean and median unique peptides/protein (the average and statistically distributed mid-value, respectively, of observed peptides uniquely matching a given protein) all increased approximately 2-fold using the *de novo* assembled *C. vulgaris* transcriptome, clearly indicative of a superior search database (Figure 3b). This identification rate marks the largest number of positive identifications for a microalgal proteomic analysis to date, and represents an order of magnitude increase compared to previously identified microalgal sub-proteomic analyses of unsequenced microalgae. Annotation of protein identifications was completed by matching to transcriptomic blastx results. Of 2,949 positive identifications, 2,660 proteins (90.2%) returned a statistically significant blast hit.

**Figure 4:**
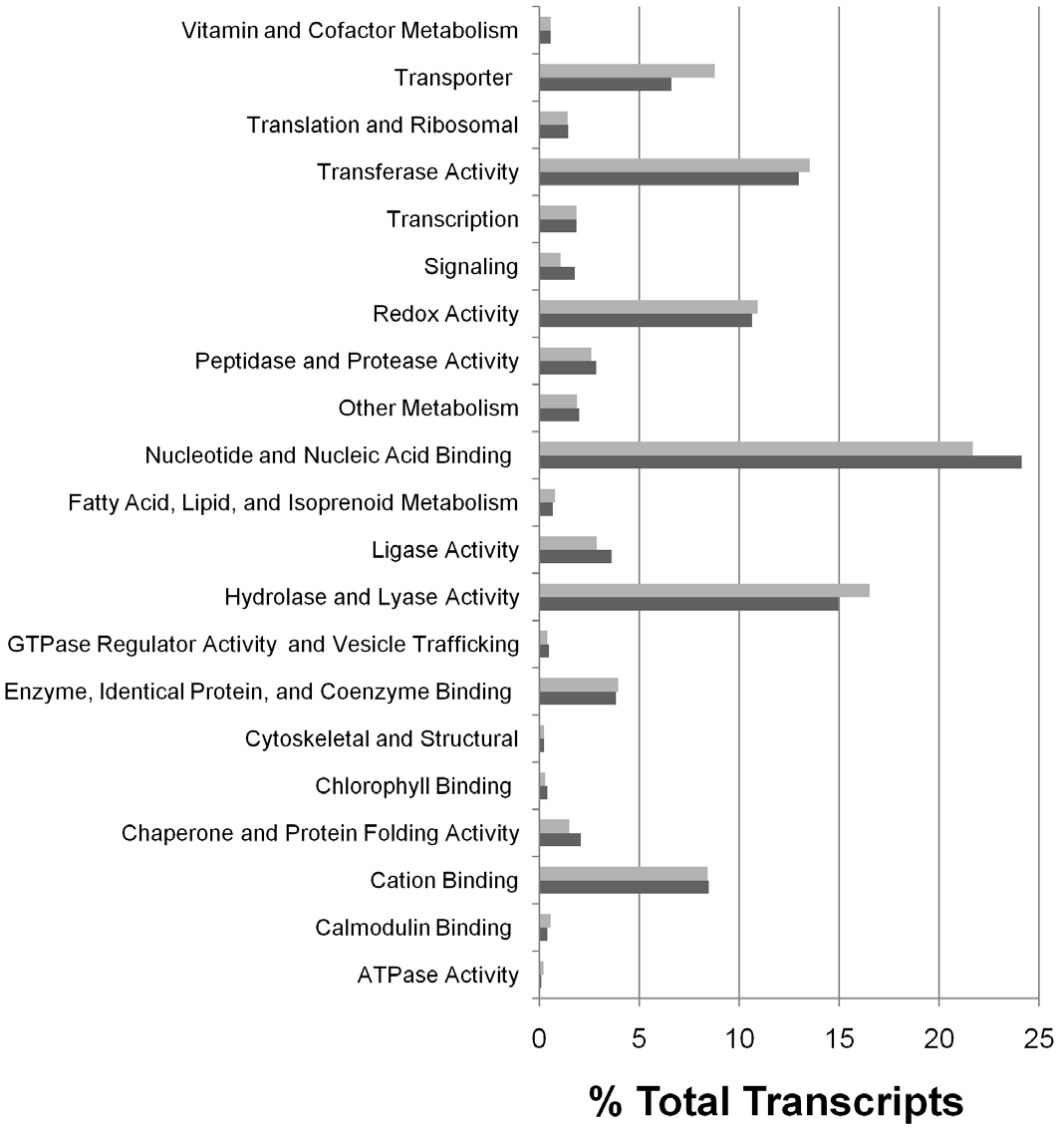
Gene Ontology enrichment analysis. Functional distribution of the complete annotated *C. vulgaris* transcriptome (grey bars) and the 2,949 transcripts corresponding to proteins identified in sub-proteomic soluble fraction via MS/MS analysis (black bars). Distribution is represented as percent of total transcripts in respective fractions.

We employed molecular function Gene Ontology (GO) enrichment analysis to assess the functional distribution of transcripts in the entire annotated *C. vulgaris* transcriptome, as well as the 2,949 transcripts corresponding to positive MS/MS identifications in the soluble sub-proteome. The results of GO enrichment are represented as the percent of total transcripts in respective fractions in Figure 4. The GO enrichment represented several categories of molecular function, with transcripts coding for nucleotide and nucleic acid binding proteins comprising the largest percentage (∼25%) of all transcripts in both the whole annotated transcriptome and the corresponding soluble sub-proteome fraction (Figure 4). Transcripts coding for proteins with transferase, hydrolase, and lyase activity were also highly enriched in both the whole transcriptome and soluble sub-proteome fraction. The gene distribution amongst the whole transcriptome and the fraction corresponding to the soluble sub-proteome fraction shows relatively equivalent percent distribution amongst the functional groupings. This result suggests a large fraction of proteins that might be expected to reside in the insoluble proteome fraction (e.g., transporters and membrane bound enzymes) were isolated by our lysis method and identified in our proteomic analysis. Indeed, this occurrence is demonstrated in the positive identification of all enzymes along the TAG biosynthetic pathway, comprised of a number of membrane-associated proteins in the endoplasmic reticulum (discussed below). The initial goal of this study was to examine the soluble proteome fraction without specific intent to examine the TAG biosynthetic components, though the identification of these components was a welcome result. The components of the FA biosynthetic pathway are expected to be largely associated with the soluble proteome fraction (not membrane-bound), and as such, the observed identification of these components was expected.

Approximately 42% of all annotated transcripts were identified in our proteomic analysis. Given the equivalent distribution across the various GO categories between the proteome and transcriptome (Figure 4), this value implies that less than half of the annotated transcribed genes from each category were identified in the proteome. However, the uniform nature of these absences suggests this is a limitation of MS/MS identification capabilities, as opposed to the systematic absence of a given class of proteins. In cases where a genome is available as a search model, identification rates for large-scale proteomic analyses are typically 35–60% [44], [45], [46], indicating the transcriptome offers a strong search database in cases where a genome is unavailable.

**Figure 5:**
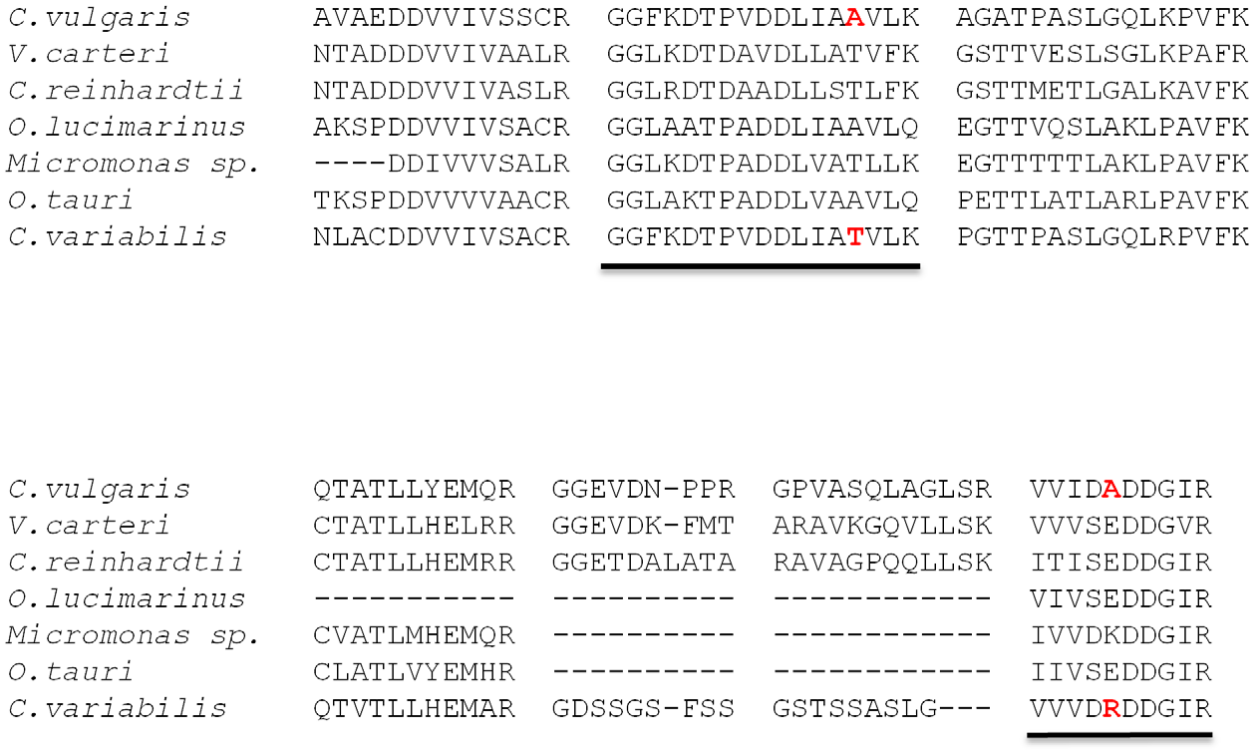
Multiple sequence alignment for acetyl-coA acyltransferase (ACAT) peptides identified via MS/MS analysis. Despite high sequence identity (Expect values<1e-124), ACAT was not identified using *Chlorophyta* sequence databases as search models in MS/MS analysis. Notably, underlined peptides differed by just a single amino acid substitution between *C. vulgaris* and *C. variabilis*, preventing positive identification. Using the *C. vulgaris* transcriptome as a sequence search database yielded 7 peptide identifications at a confidence interval >95%.

The utilization of the *de novo* assembled *C. vulgaris* transcriptome led to identification of a number of proteins along the major metabolic and biosynthetic pathways that were initially absent from the data obtained using other *Chlorophyta* sequence databases. Figure 5 provides more detail for the multiple sequence alignment of peptide fragments of acetyl-CoA acyltransferase (ACAT) identified in MS/MS analysis of *C. vulgaris* against the top seven *Chlorophyta* homologs. Despite significantly high sequence similarity (E-values<6e-124) for all homologs, Mascot searching against all *Chlorophyta* databases failed to identify ACAT. Only when utilizing the *de novo* assembled *C. vulgaris* transcriptome was ACAT identified.

**Figure 6:**
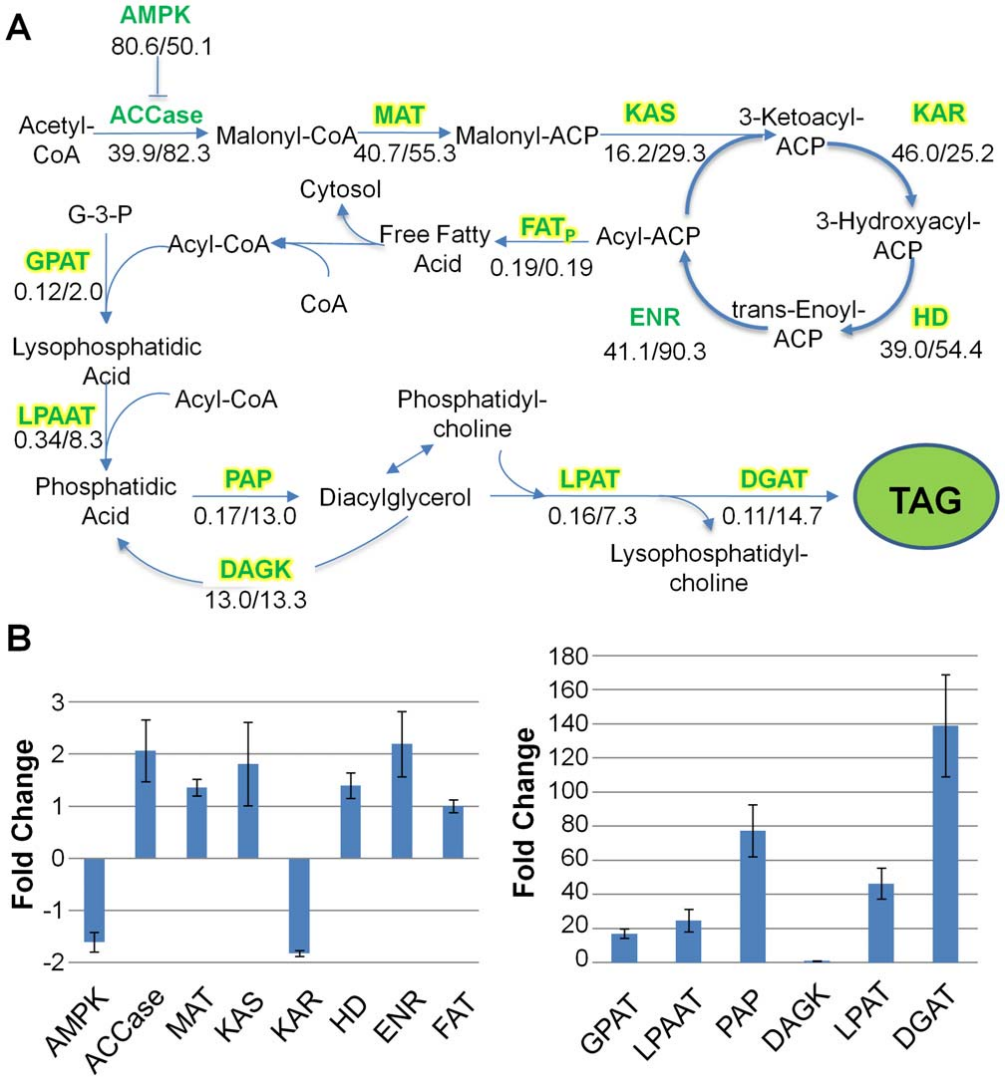
Improved pathway identification using a *de novo* assembled transcriptome database and changes in protein abundance under nitrogen depletion. (A) Critical components of the fatty acid and triacylglycerol (TAG) biosynthetic pathways were absent from initial MS/MS searches against all available *Chlorophyta* databases. Proteins highlighted in yellow were amongst the proteins absent from initial analyses, yet positively identified when searching against the *C. vulgaris* transcriptome database. Proteins in green (not highlighted) were identified using both *Chlorophyta* and *C. vulgaris* transcriptome databases. Numbers below proteins represent NSAF values (10^5^) for nitrogen replete and nitrogen deplete conditions, respectively. ACCase, acetyl-CoA carboxylase; ACP, acyl carrier protein; AMPK, AMP-activated kinase; DAGK, diacylglycerol kinase; DGAT, diacylglycerol acyltransferase; DHAP, dihydroxyacetone phosphate; ENR, enoyl-ACP reductase; FAT_P_, fatty acyl-ACP thioesterase (putative); G3PDH, glycerol-3-phosphate dehydrogenase; GPAT, glycerol-3-phosphate acyltransferase; HD, 3-hydroxyacyl-ACP dehydratase; KAR, 3-ketoacyl-ACP reductase; KAS, 3-ketoacyl-ACP synthase; LPAAT, lyso-phosphatidic acid acyltransferase; LPAT, lyso-phosphatidylcholine acyltransferase; MAT, malonyl-CoA:ACP transacylase; PAP, phosphatidic acid phosphatase. Adapted from Radakovits et al., 2010 and Hu et al., 2008. (B) Corresponding spectral count fold-changes for components of the FA (left panel) and TAG (right panel) biosynthetic components.

The utilization of our *C. vulgaris* transcriptome as a proteomic search model was also successful in identifying otherwise unidentified proteins that play critical roles in fatty acid and triacylglycerol biosynthesis (Figure 6). A significant portion of the FA pathway, including malonyl-CoA:ACP transacylase (MAT), 3-ketoacyl-ACP synthase (KAS), 3-ketoacyl-ACP reductase (KAR), and 3-hydroxyacyl-ACP dehydratase (HD) was absent from our orthologous database analysis results. The components of the TAG biosynthetic pathway, including glycerol-3-phosphate acyltransferase (GPAT), lyso-phosphatidic acid acyltransferase (LPAAT), phosphatidic acid phosphatase (PAP), lyso-phosphatidylcholine acyltransferase (LPAT), and diacylglycerol acyltransferase (DGAT) – the last of which is required for commitment into TAG biosynthesis – were also absent from the TAG biosynthetic pathway, when using orthologous search databases (Figure 6). However, these proteins were all identified in significant abundance (>10 spectral counts) using the *C. vulgaris* UTEX 395 *de novo* assembled transcriptome, indicating that they went unidentified due to lack of sequence similarity, as opposed to abundance below the limits of detection (1 spectral count). Overall, the number of statistically significant protein identifications increased nearly 2-fold when using the *de novo* assembled transcriptome as a sequence database (Figure 3b).

### Differential Protein Abundance in Fatty Acid and Triacylglycerol Biosynthetic Pathways

Chloroplastic microalgal fatty acid synthesis is proposed to occur primarily through conversion of acetyl-CoA to malonyl-CoA precursors, followed by four successive condensation reactions, ultimately resulting in the production of an acyl-ACP (Figure 6). Acetyl-CoA carboxylase (ACCase) catalyzes the first committed step of fatty acid synthesis in a two-step reaction that results in the conversion of acetyl-CoA to malonyl-CoA. ACCase inhibition via phosphorylation can be catalyzed by AMP-activated kinase (AMPK). In the next step of fatty acid synthesis, the malonyl group of malonyl-CoA is transferred to acyl carrier protein (ACP) forming malonyl-ACP in a reaction catalyzed by MAT. The subsequent series of four condensation reactions is then catalyzed by KAS, KAR, HD, and enoyl-ACP reductase (ENR). These condensation reactions ultimately lengthen precursor acyl-ACP chains by two carbons per cycle. Termination of elongation is catalyzed by an acyl-ACP thioesterase (FAT), leading to free fatty acid release and export to the cytosol, or via direct transfer of the acyl group to glycerol-3-phosphate and/or monoacylglycerol-3-phosphate in the TAG biosynthetic pathway (discussed below). For extensive review of plant and microalgal fatty acid biosynthesis, refer to Ohlrogge and Browse, 1995 and Hu et al., 2008.

Microalgal TAG biosynthesis is proposed to occur via sequential transfer of fatty acids from CoA to glycerol-3-phosphate (G3P) via the direct glycerol pathway [1], [47] (summarized in Figure 6). Fatty acid transfer to position one of G3P results in the formation of lyso-phosphatidic acid (LPA), in a reaction catalyzed by GPAT. Subsequent acyl transfer to position two of LPA leads to formation of phosphatidic acid (PA), in a reaction catalyzed by LPAAT. PA can also be formed via phosphorylation of diacylglycerol (DAG) in a reaction catalyzed by DAG kinase (DAGK) [48]. The penultimate step of TAG biosynthesis is catalyzed by PAP, resulting in dephosphorylation of PA and formation of DAG. DGAT ultimately catalyzes the final and committed step of TAG biosynthesis, in which a third acyl chain is transferred to position 3 of G3P, forming a neutral triacylglyceride [1], [49].

We examined changes in spectral counts (the total number of MS/MS spectra identifying a protein) for the components of the fatty acid and triacylglyceride biosynthetic pathways under nitrogen-replete and nitrogen-deplete conditions. Normalized spectral abundance factor (NSAF) values were utilized to calculate spectral count fold-changes, as described by Zybailov et al. [39]. Figure 6 summarizes the spectral count fold-change for the components of fatty acid and TAG biosynthetic pathways under nitrogen-deplete conditions with respect to nitrogen-replete conditions (the ratio of N-deplete to N-replete). ACCase abundance was upregulated approximately 2-fold under nitrogen-deplete conditions, while AMPK abundance was downregulated 1.6-fold. Similar to ACCase, MAT abundance was upregulated, approximately 1.4-fold, under nitrogen-deplete conditions. Enzymes catalyzing condensation reactions were also upregulated, with the exception of the approximately 2-fold down-regulation of KAR, the enzyme catalyzing the conversion of 3-ketoacyl-ACP to 3-hydroxyacyl-ACP. A protein identified via sequence alignment as a putative (non-specific) fatty acid thioesterase, proposed to terminate fatty acid elongation, displayed no change in abundance [1]. Although the absolute spectral counts for the components of the TAG biosynthetic pathway were generally lower than those of the fatty acid biosynthetic pathway (especially in the replete samples) the change in protein abundance was far more pronounced, one to two orders of magnitude greater than those observed in the fatty acid biosynthetic pathway. The corresponding *p*-values for observed changes for all components of the FA and TAG biosynthetic pathways were less than 0.05 (indicating <5% chance that the difference is random). The largest increase in abundance was observed for DGAT, with greater than 100-fold spectral count increase. DAGK showed no statistically significant change in abundance in the nitrogen-deplete state, implying that acylation of LPA is the preferred path to PA synthesis under these conditions.

## Discussion

### Chlorella vulgaris UTEX395 as a commercially relevant algal strain

Selection of suitable algal strains will be a critical step in realizing the full potential of commercial-scale photosynthetic algal cultivation for biofuel and bioproduct production. An ideal production strain will have the attributes of fast growth at acceptable cell density, cultivation robustness, and high lipid-accumulation capacity and productivity [12]. Genetic engineering, though perhaps problematic from a regulatory or community acceptance standpoint, can, at a minimum, help establish upper limits to lipid productivity and guide classical genetics or breeding programs. In addition, the glycerolipid profile of a strain will have a large impact upon the lipid's potential as a biofuel feedstock, as chain length and degree of unsaturation are critical for conversion to both biodiesel and renewable diesel and jet fuel. For example, a C18:3 fatty acid requires 7 moles of hydrogen per mole of fatty acid ester for full saturation, while C18:0 requires only 4 moles of hydrogen. As such, the glycerolipid profile of a microalgal species can ultimately have a significant impact on production costs (Robert McCormick, NREL, personal communication).

The green alga, *C. reinhardtii*, has been extensively examined as a model organism due to its ease of cultivation in a laboratory setting and its ability to be genetically manipulated. As such, investigation of *C. reinhardtii* has perhaps contributed more to the elucidation of the fundamental underpinnings of microalgal biology than any other microalga to date. However, this species lacks the intrinsic high-lipid productivity of many oleaginous microalgae. For example, under nitrogen-deplete conditions, wild-type *C. reinhardtii* starchless mutant only produces ∼20% lipid on a dry cell weight basis [12]. Conversely, the fatty acid profile, production, and accumulation capacity of *C. vulgaris* under nitrogen-deplete conditions suggests it presents an ideal feedstock for biodiesel production. Nitrogen limitation dramatically increases the desirable C18:1 fatty acid content at the expense of less desirable C18:3, C18:2, C16:1 and C16:0 content (Figure 1c). As such, nutrient-limiting conditions result in the accumulation of an oil having improved properties as a feedstock for biodiesel or renewable diesel and jet fuel. Our experimental conditions also yield ∼60% fatty acids on a dry cell-weight basis, nearly 3-fold higher than wild type *C. reinhardtii*, suggesting it is an excellent model system to examine changes in gene and protein expression under conditions that induce high-oil accumulation. We and our co-workers have had limited success reproducing the transformation techniques with *C. vulgaris* reported by Jarvis and Brown [31] and Chow and Tung [30], but we have recently improved on these methods and have developed a simple and reproducible transformation protocol (J.J. Lee and Y.-C. Chou, unpublished results). The concurrent development of systems biology and genetic tools will help establish *C. vulgaris* as a viable model organism for algal biofuels development.

### The utility of a de novo assembled transcriptome as a proteomic search model

Proteomic analysis using orthologous sequence databases presents a unique challenge in that it requires nearly identical m/z values (±1–2 Da) between the search model and peptides of interest in order to positively match an equivalent m/z ratio of statistical significance. As such, a single amino acid differential between a search model sequence and a peptide fragment sequence of interest can often result in a failure to produce a statistically significant match, leaving significant gaps in protein identification. One example of an absence caused by a single amino acid differential was observed for ACAT. Using *Chlorophyta* sequence databases, ACAT was not identified via proteomic analysis, yet was successfully identified using the *C. vulgaris* transcriptome as a search database. ACAT peptides identified via mass spectrometry using the *C. vulgaris* transcriptome were aligned against all available *Chlorophyta* ACAT sequences. Peptide sequence alignment shows just a single amino acid differential between *C. vulgaris* and *Chlorophyta* peptides in two instances, both corresponding to a species of same genus, *Chlorella variabilis*, demonstrating both the limitation of using orthologous databases for peptide identification and the advantage of using a *de novo* assembled transcriptome as a search database (Figure 5).

Improved identification capability using the transcriptome as a search database was further underscored on a more global pathway mapping scale, examining the fatty acid and TAG biosynthetic pathways. Orthologous searching identified only three enzyme components of the fatty acid biosynthetic pathway, and none of the TAG enzymatic components. Conversely, utilization of the *C. vulgaris* transcriptome as a search database allowed us to identify all enzymatic components of the fatty acid and TAG biosynthetic pathways. It is clear from these data that using the *de novo* assembled transcriptome dramatically improves proteomic identification capabilities. These results might not have been expected if the assembly or annotation of the *C. vulgaris* transcriptome was weak, or if the *Chlorophyta* database provided higher sequence identity. This is an important observation because it most notably confirms our hypothesis, and at the same time provides a measure of quality of our *de novo* assembly and annotation.

Finally, utilization of the *de novo* assembled *C.vulgaris* transcriptome allowed for identification and differentiation of critically important protein isoforms. Though no protein isoforms were identified for the TAG biosynthetic pathway, homomeric and heteromeric ACCase isoforms, as well as multiple KAS isoforms, were identified during the annotation stage. Isoform differentiation can have a dramatic impact upon strain engineering strategies. For example, it has been suggested that overexpression of cytosolic homomeric ACCase, coupled with plastidial sub-cellular localization, as opposed to overexpression of the more complex, multi-subunit heteromeric plastidial isoform, may be a simpler and more efficient means to increase fatty acid content in oleaginous organisms [50]. Targeted strain improvement efforts and complete pathway analyses will thus be greatly facilitated by the isoform identification and maximal identification coverage a *de novo* assembled transcriptome search database affords.

The gene ontology analysis was encouraging because it indicated that all classes of proteins were equally represented in the proteome in comparison to the transcriptome, including proteins that would be expected to reside mainly in the insoluble fraction. But it is also a warning that the majority of transcribed genes were not found in the proteome. It is likely that this failure to identify most transcribed gene products is due to some combination of quantitative limits to the GeLC-MS methodology and to post-transcriptional regulation. The distinction between these two possibilities can have a major impact on the quantitative proteomic analysis, especially as it would be applied to hypothesis-driven strain improvement programs and will need to be evaluated on a gene-by-gene basis for pathways of interest. Future work involving quantitative transcriptomic analysis, insoluble proteomic analysis, and more focused searches for specific missing proteins will help shed light on this missing piece of the puzzle.

### Changes in Fatty Acid and TAG Biosynthetic Pathways in a High-Lipid State

As observed previously for *C. cryptica* [51], our analysis also found upregulation of ACCase under lipid-accumulating conditions. More importantly, though, our current work also established that the majority of the other fatty acid biosynthetic pathway components are upregulated under nitrogen-depletion and concurrent lipid accumulation. Interestingly, AMPK was down-regulated under high-lipid producing conditions. AMPK is proposed to serve as a fatty acid beta-oxidation “metabolic master switch,” acting as a direct ACCase inhibitor (and indirect carnitine palmitoyltransferase (CPT-1) activator in higher eukaryotes) [52]. This lends potential insight into the regulation of fatty acid synthesis through rate-limiting ACCase activity and concurrent increase in beta-oxidation. Overexpression of the ACCase gene in both *C. cryptica* and *N. saprofila* failed to significantly increase lipid accumulation [53]. A number of mechanisms have been proposed to explain this observation, including post-transcriptional regulation and feedback inhibition. It is possible AMPK also played a critical role in driving the equilibrium between acetyl-CoA and malonyl-CoA in the reverse direction, ultimately slowing the rate of fatty acid biosynthesis and increasing the rates of fatty acid beta-oxidation. The activity of AMPK under nitrogen-replete and nitrogen-deplete conditions warrants further investigation.

The possibility must be considered that our decision to harvest cultures at the onset of stationary phase yielded cells that were past their peak in abundance of the enzymatic components of the fatty acid biosynthetic pathway (even though many significant increases were observed). Resultant late stage down-regulation of synthesis may explain the decrease observed for KAR under nitrogen-limited conditions. As synthesis of fatty acid biosynthetic machinery is shut down, protein turnover is likely to occur, as cells may be under a state of high catabolic activity in order to recycle internal nitrogen stores. However, protein abundance is not directly reflective of protein expression rates or activity. Thus, it is possible that KAR enzymatic rates remain unchanged despite decreased abundance. It is also unclear whether the increased abundance of fatty acid biosynthetic components (and decrease in KAR abundance) is due to altered rates of mRNA expression and translation, or mRNA and protein turnover. A more complete integrated systems biology analysis, incorporating transcriptomic, proteomic, and metabolomic data will be necessary to fully elucidate potential flux bottlenecks in the fatty acid pathway.

Our results demonstrate that TAG biosynthetic machinery abundance is upregulated significantly higher than the fatty acid synthesis machinery in a high-lipid accumulation state. This massive upregulation can again likely be attributed to the late-stage harvest of nitrogen-deplete cells. At this stage, cells have neared stationary phase, having exhausted internal nitrogen stores. Photosynthetic energy and carbon fixation can continue (albeit with presumably altered efficiency indicated by the reduction in pigmentation in the starved, chlorotic cells, Figure 1a). The most effective diversion of the fixed carbon and reducing equivalents generated by photosynthesis is their conversion to TAGs (as reflected in fluorescence imaging of neutral lipids, Figure 1d). As observed in *C. reinhardtii* lipidomics analysis, acyltransferases were amongst the most abundant lipid droplet-associated proteins observed [11]. As such, with the intracellular space largely encompassed by neutral lipids, it is to be expected that we would observe significant abundance of TAG-related acyltransferases. The dramatic differential between fatty acid biosynthetic and TAG biosynthetic components may imply TAG biosynthesis may also play a significant role in the rate-limiting production of neutral lipids, suggesting future studies aimed at strain improvement might be focused upon overexpression of TAG biosynthetic components in addition to fatty acid biosynthetic components.

Notably, none of the TAG biosynthetic machinery was identified under nitrogen-replete conditions, suggesting TAG biosynthesis is either largely in an “off state” or at very low levels in early growth phase, and the majority of the 10% fatty acids observed under nitrogen-replete conditions is derived from structural membrane phospholipids. Interestingly, we observed steady-state abundance of DAGK between nitrogen-replete and nitrogen-deplete conditions. DAGK catalyzes the conversion of DAG to PA via phosphorylation of DAG, and its activity has been shown to increase upon activation of the phosphoinositide (PI) pathway. It is, therefore, proposed to function as a termination cue in the formation of DAG [48]. Steady state abundance may be indicative of steady-state activation of the PI pathway, potentially pointing to a minimal baseline phospholipid production level, required for cell viability.

We hypothesize that future analyses using intermediate harvest points will lead to a less pronounced differential between fatty acid and TAG biosynthetic components, with an increased abundance of fatty acid components and a decrease in abundance of TAG components prior to nitrogen exhaustion. Future analyses will therefore be focused upon intermediate accumulation, which will allow for abundance mapping throughout the lipid accumulation cycle and help clarify the rates of TAG component expression. Concurrently, quantitative analyses of PA, DAG, and TAG will lend further insight into the flux through the TAG pathway, as well as temporal regulation throughout the lipid accumulation cycle.

### Conclusions

The prevalence of microalgal translational gene regulation necessitates higher-level omic analyses at the protein level in order to fully elucidate changes in gene expression under varying conditions. However, proteomic analysis of unsequenced microalgae is clearly limited by the lack of flexibility in fragment matching. Our results underscore how much more powerful proteomic analysis can be when accurate sequence information is available, and demonstrate the utility of a *de novo* assembled transcriptome as a search model for proteomic analysis of unsequenced microalgae. Strain improvement strategies targeting increased lipid accumulation and productivity as well as improved understanding of the relevant basic biology will be critically enhanced by utilization of our transcriptomic sequence data combined with proteomic abundance data. We have focused our initial investigation of differential protein expression upon dramatically different lipid accumulation states (10% vs. 60% fatty acid) in N-replete and deplete *C. vulgaris*. These analyses indicate that the fatty acid and TAG biosynthetic pathways are dramatically upregulated (TAG>fatty acid) under nitrogen limitation. Data from intermediate accumulation states will likely provide a wealth of additional information with regards to the stages at which gene and protein-expression are initiated. Carbon flux analyses, glycerolipid speciation, and metabolomic analysis will ultimately need to be initiated to complement comparative transcriptomic and proteomic analyses, in order to fully assess flux through lipid-relevant pathways of interest on a comprehensive systems biology level.
